# A short humorous intervention protects against subsequent psychological stress and attenuates cortisol levels without affecting attention

**DOI:** 10.1038/s41598-021-86527-1

**Published:** 2021-03-31

**Authors:** Eva Froehlich, Apoorva Rajiv Madipakkam, Barbara Craffonara, Christina Bolte, Anne-Katrin Muth, Soyoung Q. Park

**Affiliations:** 1 Decision Neuroscience and Nutrition, German Institute of Human Nutrition (DIfE), Arthur-Scheunert-Allee 114-116, 14558 Nuthetal, Germany; 2 Institute for Psychology, University of Lübeck, 23562 Lübeck, Germany

**Keywords:** Psychology, Health care

## Abstract

Presentation of humor simultaneously with a stressful event has been shown to dampen the psychological and physiological responses of stress. However, whether a relatively short humorous intervention can be utilized to prevent the subsequent stress processing is still underinvestigated. Furthermore, it is unknown, whether such a humor intervention changes stress processing at a cost of cognitive functioning. According to the broaden-and-build theory inducing positive emotions may subsequently impact cognitive performance. Here, we investigated whether humor protects against subsequent stressors by attenuating both, psychological and physiological stress levels and whether this affects cognitive performance. Participants watched either a humorous or a neutral movie, underwent stress induction and performed in a visual search task. Compared to the control group, psychological stress levels and salivary cortisol levels were lower in the humor group, yet no differences were found in response times and accuracy rates for the visual search task. Our results demonstrate that a short humorous intervention shields against subsequent psychological stress leaving cognitive performance intact, thus making it highly applicable to improve mental and physical health in everyday life situations.

## Introduction

Picture yourself on the train on the way to an important job interview or presentation. You know you will be faced with a stressful situation, in which you have to perform to the best of your abilities. To distract yourself from the upcoming event and possibly to kill some time on the commute, you might grab your phone and start watching funny movie clips. Yet, the consequences of this seemingly banal action could be more far-reaching than expected. Is it possible, that this short humorous intervention is not only entertaining but possibly protects against the subsequent stressful situation? And will it have an effect on your cognitive performance by affecting your attention?

Stress has been identified to be one of the largest risk factors for public health. It can affect individuals psychologically as well as impact the nervous, the endocrine and the immune system^1^. A number of observational as well as laboratory studies have focused on the relationship of humor and stress and whether humor can offset the adverse effects of stress. In these studies, humor has often been operationalized in terms of trait humor (sense of humor) or in terms of an instantaneous humorous intervention. Stress, on the other hand, has been either assessed by subjective reports (psychological stress) or by biomarkers, such as heart rate or skin conductance rate (physiological stress). For instance, Abel^2^ found individuals with higher self-reported sense of humor to experience less stress and state anxiety than those with lower scores. Similarly, Martin & Dobbins^3^ observed sense of humor to moderate the adverse effects of daily stressors on secretory immunoglobulin, an index of immune functioning. In contrast, when stress is experimentally induced, sense of humor does not seem to affect the psychological and biological stress response, yet a humor intervention does^4,5^. Subjects score lower in state anxiety after watching a funny film compared to an informative one^6^ and this seems to be more effective when the humorous clip precedes the unpleasant stimulus^4^. In the absence of stress induction, state anxiety decreases after viewing a funny film compared to a hopeful or sad one^7^. Similarly, humor generation (i.e., producing a humorous narrative to a stressful film) has been found to moderate the effects of, both psychological and physiological stress more reliably than generating an informative narrative^5^. However, Rizzolo and colleagues^8^ observed merely a reduction in the physiological markers of stress (i.e., heart rate and blood pressure) after a 30-min humorous intervention but report no effects on daily stressors scores, i.e., on psychological markers. Taken together, the evidence seems to point towards a beneficial effect of humor on stress, either directly or as a moderator. However, in most studies the humorous intervention occurs simultaneously to stress-inducing measures^5^ or in succession^6 but 4^, is rather time-consuming^8^ and stress is primarily assessed on the psychological level^3,4 but 5^. Therefore, it still remains an open question, whether a short humorous intervention suffices to counteract the adverse effects of both psychological and physiological stress even before the stressful event occurs. In other words, will a humorous intervention (compared to a neutral one) preceding an experimentally induced stressor lower psychological and physiological stress levels?

Several potential mechanisms have been proposed with respect to the beneficial effects of humor on health and stress, each assigning a somewhat different function to humor^9^. For instance, humor is thought to moderate the adverse effects of stress by cognitively reassessing stressful events. It may be used to distance oneself from stressful situations and to reinstate the feeling of being in charge. Thus, it facilitates adaptive and effective coping^9,10^. Another possible mechanism is via the inducement of positive emotions and states, that attenuate negative emotions, such as anxiety, depression and other stress-related health issues^9,11^. According to the broaden-and-build theory^12,13^ these positive emotions impact cognitive performance as they affect the thought-action repertoire. More specifically, the broaden-and-build theory states that positive emotions, such as joy, expand the scope of attention whereas negative emotions, such as anxiety, narrow it. Accordingly, a number of studies using visual attention tasks have provided evidence that subjects tend to focus on local features of visual stimuli when being in a negative mood compared to focusing on global, configural features when being in a positive mood^14,15,16,17^. Taking these findings into account, it remains an open question whether the positive emotions induced by humor not only attenuate stress but consequently impact cognitive performance related to attentional processes.

The aims of this study were twofold. First, we aimed to investigate whether a short humorous intervention reduces subsequent psychological and physiological stress. Second, we tested whether this beneficial effect affects cognitive performance in the form of attention. To do so, we randomly assigned participants into two groups: a humor and a control group. Humorous or neutral moods were induced by means of short films (approx. 9 min) since they have been shown to be most effective in doing so^16,18^. After watching the clips, participants were asked to rate the films’ funniness. Participants were then subjected to a stress test. Stress was operationalized by having to perform unforeseen subtractions while participants were exposed to randomly delivered aversive electric shocks. Prior to the stress test, the intensity of the shocks was adjusted to match the individual’s level of pain tolerance. Before watching the video clips and after the stress test, participants indicated their subjective stress level. Additionally, four salivary cortisol samples were obtained before and during the course of the experiment which served as the physiological marker of stress^1^. Finally, a visual search task was used to evaluate the influence of humor on (visual) attention. Participants were shown different arrays of Greebles, and had to point the odd one out. Greebles are artificial objects initially created for investigating face recognition. Each Greeble belongs to one of five families defined by the shape of protruding appendages and has one of two genders determined by an up or downward pointing central part^19^. We choose this set of stimuli deliberately, as (similar to faces) one cannot solely rely on local shape features to successfully identify Greebles but has to process and integrate configural information as well^19,20,21,22^. To control for possible group differences in state and trait anxiety as well as sense of humor, subjects also completed the State-Trait-Anxiety Inventory (STAI)^23^ and the Sense of Humor Scale^24^, respectively at the very end of the experimental session. We hypothesized that if humor protects against subsequent aversive effects of stress, then we should observe lower psychological and physiological stress levels (i.e., a faster decrease in cortisol levels) in the humor group compared to the control group. Furthermore, in line with the broaden-and-build theory, if being in a humorous mood promotes the broadening of attention and the processing of global rather than local features, we expected the humor group to be more attentive to global, configural features and thus identify Greebles faster and more successfully than the control group. Therefore, reaction times and/or error rates in the visual search task should be lower in the humor group compared to the control one.

## Results

### Funniness ratings

As a first step, we assessed whether the humorous film was indeed perceived to be funnier than the neutral one by comparing the funniness ratings of the humor group with those of the control group. We observed a significant difference in funniness ratings, *W* = 1485.5, *p* < 0.001, *r* = − 0.80 with the humor group rating their film funnier than the neutral one, *Mdn* = 7.00 and *Mdn* = 2.00, respectively (see Table 1).

**Table 1:** Group characteristics including psychological and physiological measures as well as behavioral data for the humor and the control condition.

	Humor		Control	
	*M* (*SD*)	*Mdn* (range)	*M* (*SD*)	*Mdn* (range)
**Psychological measures**				
Sense of humor	29.0^a^ (5.90)	28.1^a^ (20–44.5)	27.7^b^ (5.20)	27.5^b^ (19.6–46)
Funniness ratings	6.22^a^ (1.59)	7.0^a^ (2–9)	2.37^b^ (1.22)	2.0^b^ (1–6)
Anxiety (state)	43.5^a^ (7.67)	42.0^a^ (32–68)	41.1^b^ (9.51)	37.5^b^ (24–60)
Anxiety (trait)	48.0^a^ (2.41)	48.0^a^ (43–53)	47.4^b^ (2.83)	47.5^b^ (40–53)
Stress (pre)	3.88^c^ (1.70)	3.0^c^ (1–7)	3.37^d^ (2.01)	3.0^d^ (1–7)
Stress (post)	5.80^c^ (1.83)	6.0^c^ (2–8.5)	6.67^d^ (1.64)	7.0^d^ (4–9)
Stress (change)	1.93^c^ (1.61)	2.0^c^ (− 1 to 5)	3.31^d^ (2.18)	3.3^d^ (− 2 to 8)
**Physiological measures**				
Pain level	0.18^a^ (0.13)	0.14^a^ (0.05–0.56)	0.12^b^ (0.07)	0.10^b^ (0.05–0.43)
T1 cortisol	6.60^e^ (7.16)	3.91^e^ (0.35–28.5)	5.47^b^ (4.17)	4.17^b^ (0.25–17.7)
T2 cortisol	5.40^e^ (5.42)	3.64^e^ (0.45–24.4)	5.63^b^ (4.80)	4.58^b^ (0.47–24.6)
T3 cortisol	4.60^e^ (4.56)	2.92^e^ (0.11–19.6)	5.09^b^ (4.71)	3.56^b^ (0.29–25.2)
T4 cortisol	3.53^e^ (2.77)	2.75^e^ (0.71–11.8)	3.70^b^ (2.85)	2.68^b^ (0.55–12.7)
AUC_i_ (T1–T4)	− 134.3^e^ (220)	− 60.1^e^ (− 781 to 171)	− 43.8^b^ (163.5)	− 34.9^b^ (− 463 to 447)
AUC_i_ (T2–T4)	− 59.4^e^ (91.9)	− 38.9^e^ (− 416 to 80.9)	− 53.8^b^ (88.0)	− 48.2^b^ (− 276 to 104)
**Behavioral measures**				
Response times (sec)	3.10^f^ (0.37)	3.14^f^ (2.17–3.86)	3.06^f^ (0.41)	3.03^f^ (2.18–3.83)
Accuracy rates (%)	85.5^f^ (8.18)	87.2^f^ (60–97.2)	87.6^f^ (9.10)	91.1^f^ (61–98.3)

*M*  mean, *SD*  standard deviation, *Mdn*  median, *T1–T4*  time points 1–4, *AUCi*  area under the curve with respect to increase.

^a^*n* = 41.

^b^*n* = 38.

^c^*n* = 20.

^d^*n* = 19.

^e^*n* = 40.

^f^*n* = 37.

### Humor protects against stress on the psychological level and attenuates cortisol levels

**Figure 1:**
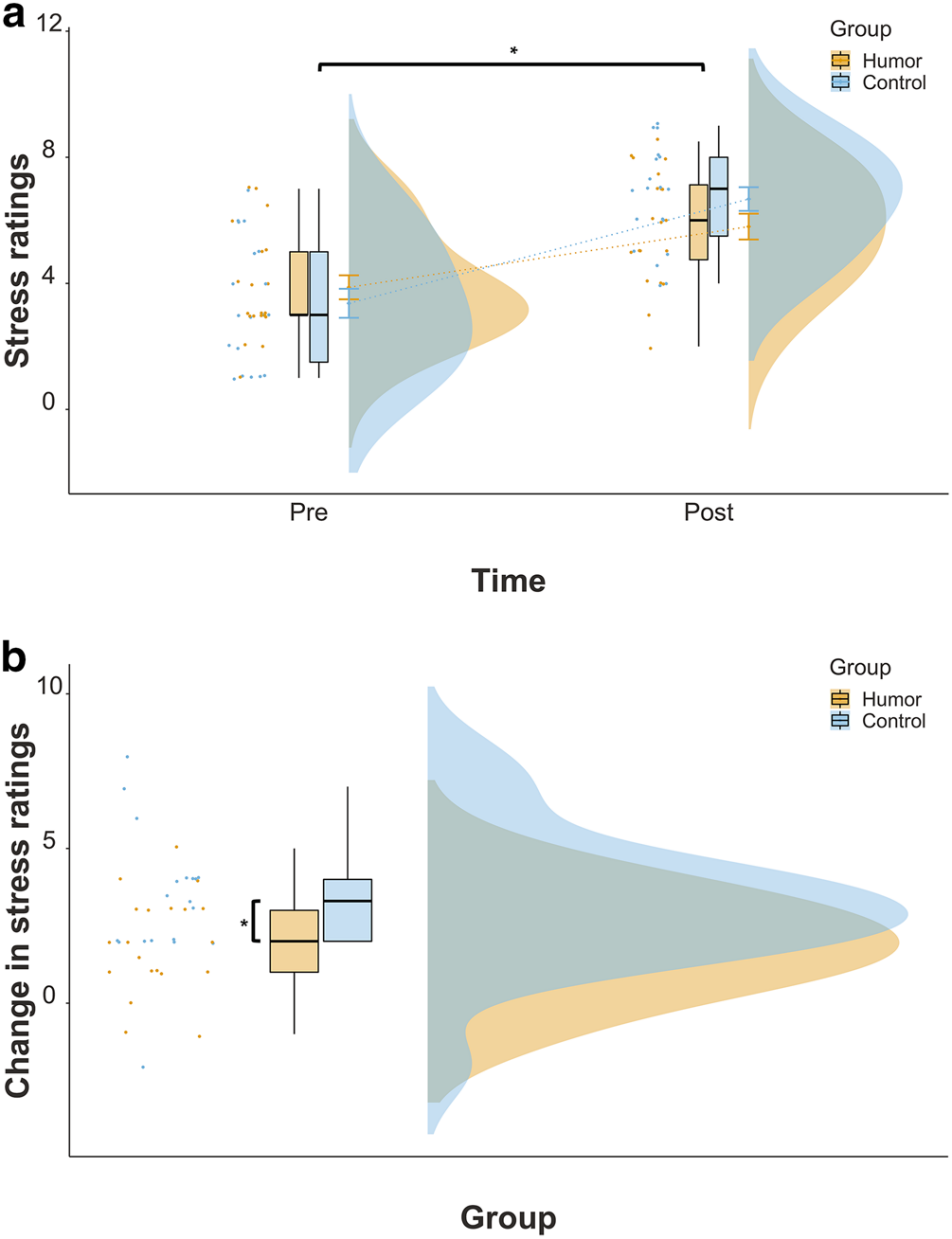
**(a)** Psychological stress levels before and after the stress test as a function of group. Asterisks (*) indicate statistically significant differences: *p < .05. Error bars denote the standard error of the (group) mean. **(b)** Change of psychological stress levels as a function of group. To measure the change in psychological stress levels, the difference between stress ratings pre- and succeeding the stress test was calculated. Asterisks (*) indicate statistically significant differences: *p < .05.

Next, we investigated the protective effect of humor on psychological and physiological stress levels. First, we performed a mixed effect ANOVA with group as a between and time as a within subject factor to test the group effect on psychological stress. Due to a mishap during data transfer, we lost psychological stress ratings for 40 subjects, so that data from only 39 participants (humor group: *n* = 20) was available for analyses. We found a significant effect of time, *F*(1, 37) = 73.2, *p* < 0.001, *η*^*2*^ = 0.36 with larger subjectively reported stress scores after the stress test (6.23) than before (3.63). More importantly, we found a significant interaction between group and time, *F*(1, 37) = 5.10, *p* < 0.05, *η*^*2*^ = 0.04. Although both groups reported an increase in perceived stress, it was less for the humor compared to the control group (5.80 vs. 6.67; Fig. 1a). Accordingly, the change in psychological stress was also significant, *W* = 108, *p* < 0.05 (*Md* = 2.00 and 3.30 for the humor and control group, respectively; Fig. 1b), *r* = − 0.41.

Next, we tested group differences in physiological stress with an emphasis on the change in cortisol levels over time. Similar to the psychological stress analysis, we performed a mixed effect ANOVA with group as a between and time as a within subject factor. We included three measurement time-points to account for baseline cortisol levels before the experimental manipulation (i.e., before watching the video clip; T1), before pain level assessment and stress induction (T2) as well as right after stress induction (T3)^e.g.^^25^. We found a significant main effect of time, *F*(1.20, 91.2) = 8.86, *p* < 0.01, *η*^*2*^ = 0.009 and, more importantly, a significant interaction of time and group, *F*(1.20,91.2) = 4.66, *p* < 0.05, *η*^*2*^ = 0.005. Follow-up analyses showed that only for the humor group cortisol levels declined significantly from T1 to T2 as well as from T1 to T3, *t*(152) = 3.02, *p* < 0.05, *r* = 0.24 and *t*(152) = 5.04, *p* < 0.001, *r* = 0.38, respectively (cf. Table 1 for means). Further inspection indicated, that this decline from T1 to T3 was significantly larger than that from T1 to T2, *t*(152) = 3.02, *p* < 0.01, *r* = 0.24. Moreover, we found a marginally significant difference in cortisol levels between T1 and T3, *t*(152) = 2.84, *p* = 0.06, *r* = 0.22 with a larger effect for the humor group compared to the control one (2.0 and 0.38 respectively). No further comparisons of interest were significant, all *p*’s > 0.11. All follow-up tests were corrected for multiple testing.

**Figure 2:**
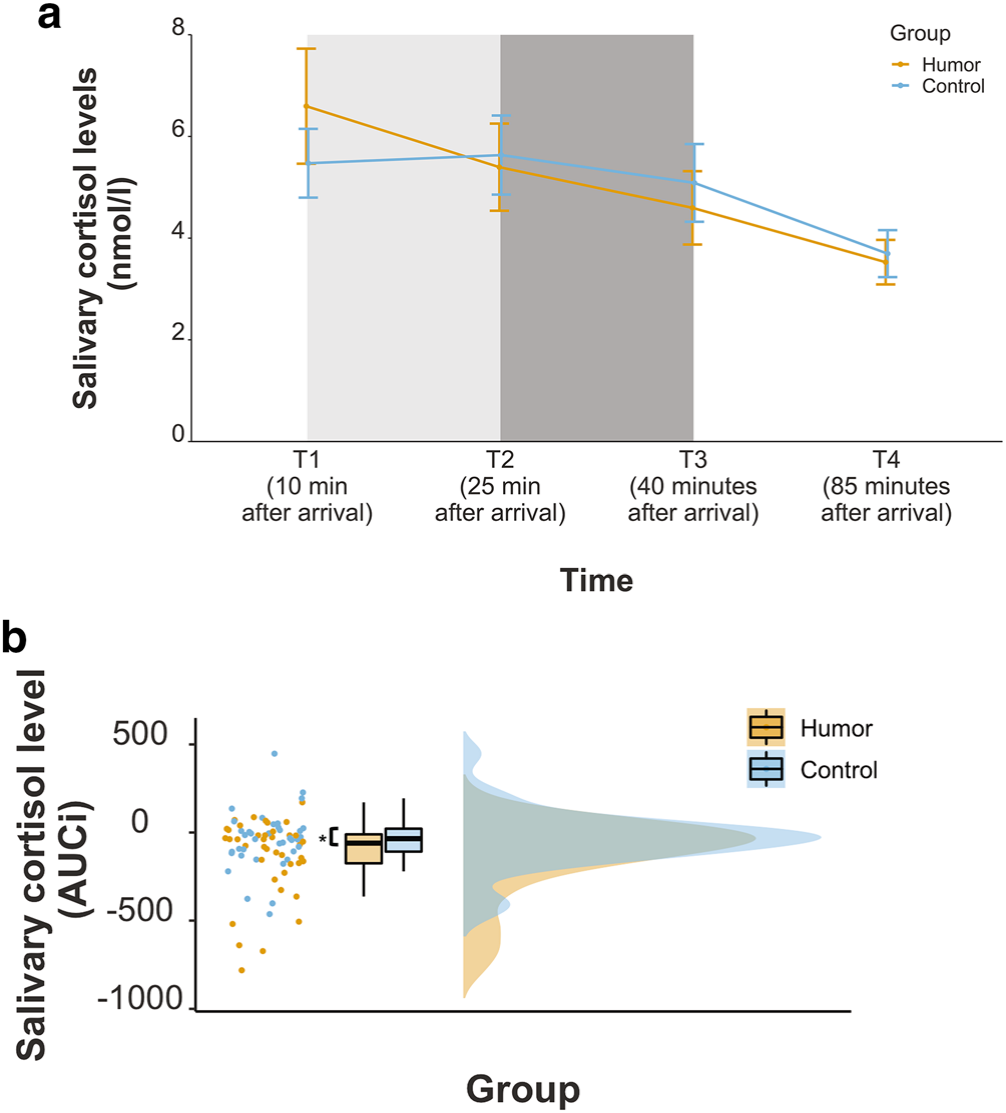
**(a)** Salivary cortisol levels over the course of the experiment as a function of group. Depicted are raw nmol/l cortisol values for all four measurement timepoints. The light grey shaded area indicates the humor induction phase, the dark grey shaded area indicates the pain tolerance assessment and the stress induction phase. Error bars denote the standard error of the (group) mean. **(b)** Change of physiological stress levels over the course of the experiment as a function of group. To assess the change in physiological stress levels, the area under the curve with respect to increase was calculated including all four measurements of cortisol levels over the course of the experiment. Asterisks (*) indicate statistically significant differences: *p < .05.

We observed a decline in cortisol levels for the humor group after the experimental manipulation and during the stress induction phase and even though this effect was marginally more pronounced for the humor group than for the control group, we did not observe a raise of cortisol levels from T2 to T3 as would have been expected given the successful induction of psychological stress. As illustrated in Fig. 2a and Table 1, cortisol levels in both groups were lower after the stress induction phase than before, therefore, we checked whether cortisol levels were more attenuated per se for the humor compared to the control group. To do so, we computed the area under the curve with respect to increase as a function of time (AUC_I_)^26^ taking all four measurement time points into account. An independent t-test revealed a significant difference in cortisol levels between the humor and the control group, *t*(71.3) = − 2.091, *p* < 0.05, *r* = 0.24. The humor group was found to have lower cortisol levels (i.e., AUC_I_) than the control group (-3.13 and − 1.00, respectively) indicating a greater decrease in salivary cortisol and therefore, possibly in physiological stress levels within that group (Fig. 2b). Interestingly, we observed no significant correlation between the changes in psychological stress levels and the decrease in physiological ones, *p* > 0.48.

**Figure 3:**
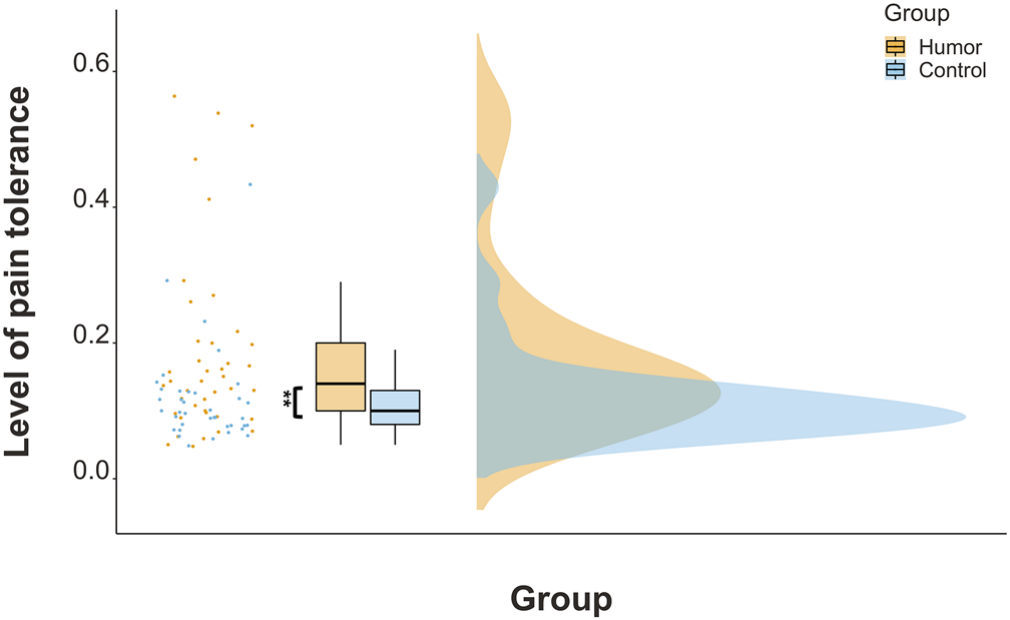
Pain tolerance as a function of group. Asterisks (*) indicate statistically significant differences: **p < .01.

As the intensity of the aversive electric shocks during the stress test was adjusted according to each individual’s pain tolerance, we additionally tested whether humor enhanced pain tolerance levels^9^. An independent t-test with group as the grouping variable showed a significant difference in the level of pain tolerance between the humor and the control group, *t*(61.6) = 2.79, *p* < 0.01, *r* = 0.33. Those who watched the humorous clip showed higher levels of pain tolerance than those who watched the neutral one, 0.18 and 0.12, respectively (Fig. 3). In line with that observation, funniness ratings correlated significantly with pain tolerance across all participants, *r*_*τ*_ = 0.19, *p* < 0.05. The funnier the clip was rated, the higher the pain level the participants tolerated.

### Humor does not impact subsequent cognitive performance

Subsequently, we investigated whether humor impacted cognitive performance in the form of attention. For that purpose, we calculated an independent t-test for response times and a Wilcoxon rank-sum test for accuracy rates from the visual search task as a function of group. We found neither an effect of group on response times, *t*(71.5) = 0.44, *p* > 0.60 nor on accuracy, *W* = 548, *p* > 0.93. To investigate whether the non-significant results allow for the conclusion, that subjects in the control condition performed equally well to those in the humor condition, we conducted two additional Bayesian t-tests. Both Bayes factors (BF) indicate moderate to strong evidence for the null hypotheses with BF_0-_ = 5.62 for response times and BF_0+_  = 9.49 for accuracy rates. In other words, the response time data are 5.62 times more likely to occur under the null hypothesis than under the alternative hypothesis, for the accuracy data this is 9.49 times as likely^27,28^. However, since humor was found to significantly impact changes in psychological stress and cortisol levels, we further investigated whether these changes would have an effect on cognitive performance. To do so, response times and accuracy data from the visual search task were subjected to two separate regression analyses. The regression models included the change in psychological stress and cortisol levels as predictors as well as their interactions. The change in psychological stress was calculated by subtracting the stress ratings before the stress test from those after the stress test. The AUC_I_ represented the change in cortisol levels. Again, the results showed no reliable effect on response times, *p* > 0.53 or accuracy data, *p* > 0.996.

### Sense of humor and anxiety scores

We performed three separate Wilcoxon rank-sum tests to exclude possible group differences in the sense of humor as well as trait and state anxiety. There were no significant differences between the two groups in sense of humor, *p* > 0.39; as well as trait anxiety, *p* > 0.35. Also, no significant group differences were observed for state anxiety, *p* > 0.93.

### Individual differences as a function of perceived funniness

Finally, we explored whether individual funniness ratings predicted psychological stress as well as changes in cortisol levels, pain tolerance, and visual search task performance (i.e., response times and accuracy). To do so, we performed bivariate correlations between funniness ratings and each of these variables within each group. These analyses aimed to investigate if there is an explicit link between the perceived funniness of the films and experimental outcomes. After correcting for multiple testing, the only significant association was a negative correlation between funniness ratings and accuracy within the humor group, *r*_*τ*_ = − 0.29, *p* < 0.05. Those who perceived the film as funnier, showed lower accuracy rates.

## Discussion

Here, we aimed to investigate the protective power of a short humorous intervention against psychological and physiological stress and its subsequent impact on cognitive performance, more specifically on attention. With respect to the first research question, our results confirm the hypothesized beneficial role of humor. Watching funny video clips attenuates psychological stress as well as cortisol levels in the absence of evidence of physiological stress. These findings are in line with previously reported ones, although the current study differs in three important aspects from earlier research. First, we specifically targeted the question whether humor could shield against future stress. This required the humorous intervention to naturally precede the stressful event. In previous studies stress induction usually took place before the humorous intervention^6^ or concurrently^5 but 4^. Second, we put emphasis on a very short intervention that is easily applicable in everyday life. Third, most studies investigating humor and stress assess stress solely psychologically, i.e., subjects were asked for anxiety ratings or ratings on daily stressors or hassles^3,6^. Only some of these studies additionally obtained data on physiological stress, with an emphasis on markers affecting the nervous system, such as heart rate, skin conductance response or blood pressure (or a combination of those)^5,8^. They consistently reported a decrease in these variables in the humor condition, however, only Newman & Stone^5^ induced a specific physiological stress response whereas Rizollo and colleagues^8^ looked at general physiological stress levels. By observing an attenuation of cortisol levels in the humor condition, we found a similar decrease in physiological stress markers, yet despite successfully evoking psychological stress, we were not able to provide evidence for a physiological stress response. This may be less surprising than it seems. Firstly, perceived stress and physiological stress levels can be only little to moderately associated given the complexity of the neurobiological processes involved^29^. Secondly, our measurement time points of salivary cortisol were chosen in accordance with other studies investigating physiological stress responses^e.g.^^25^ and to examine whether cortisol levels from the humor group return faster to baseline levels than those of the control group. Therefore, we might have missed the optimal time point to measure peak cortisol levels, which possibly explains why we did not observe an increase in physiological stress levels following the stress induction phase. Similar to the decrease in physiological stress markers, Martin & Dobbins^3^ found sense of humor to moderate immunosuppressive effects of stress. In the present study, physiological stress was operationalized by inspecting the change in salivary cortisol levels. Cortisol has been proven to reliably indicate stress levels^1,30^, and has been called the “method of choice” when assessing cortisol effects in stress research^29^. A combination of markers tapping into the nervous, the immune as well as the endocrinological stress responses would allow future research, for instance via latent change modelling, to even further specify the impact of humor on the specific physiological stress markers.

Interestingly, we found no group differences in trait anxiety and sense of humor, indicating that the observed effects on psychological stress and cortisol levels are rather likely driven by the humorous vs. control intervention. Contrary to previous studies, we did not observe group differences in state anxiety, which could be due to different experimental designs, particularly due to the order of stressful and humorous events^6^ or to the absence of a stress induction phase^7^. In line with that thought, participants of the present study completed the questionnaires at the end of the study, i.e., there was a considerable lapse of time compared to previous studies. However, how precisely timing affects the relationship of humor and stress should be subject to further research.

Our results show that humor protects against psychological stress and effectively attenuates salivary cortisol levels, however, it is still unclear why participants may benefit from a humorous intervention. Although our study was not explicitly designed to investigate the underlying mechanisms systematically, a closer examination of our results regarding pain tolerance might give a hint. We observed higher levels of pain tolerance in the humor compared to the control group thus replicating earlier results^9,31^. Yet, comparable findings have been reported when subjects saw sad or dramatic clips, or listened to their favorite music. Therefore, these authors concluded, that it is not the humorous intervention per se, that has the beneficial effect, but rather the distraction it provides^10^. Additionally, since in our design the humorous intervention precedes the stressful event, carry-over effects may also play an important role. Furthermore, we do not know whether and how participants actively utilized humor to strategically cope with the stressor. Within the frame of the present study, we can neither conclusively confirm or negate the distraction hypothesis, the possibility of carry-over effects nor the active utilization of humor to build up resilience. Yet, it is conceivable, that the protective effect of humor on stress is based on similar mechanisms. Nonetheless, even though psychological stress and cortisol levels are reduced by humor, the underlying neural and cognitive mechanisms may still be different. Consistent with previous research reporting only moderate associations between the two kind of stress levels^29^, the change in psychological and physiological did not correlate in the present study. Future research is needed to not only further investigate the underlying mechanisms of the beneficial effect of humor on stress but also to discern whether humor impacts psychological and physiological stress in a similar manner.

Regarding the second research aim of the paper and contrary to our expectations, we found no reliable effect of humor on attention, neither in response times nor in accuracy rates. Likewise, further analyses investigating possible effects of changes in psychological and salivary cortisol levels due to the beneficial effects of humor yielded null effects as well. As positive emotions have been found to broaden the scope of attention in global–local visual attention tasks, we hypothesized to observe a similar effect in the humor group compared to the control one^16^. What we have found instead, is a successful attenuation of psychological stress and cortisol levels in the humor group without simultaneously impacting cognitive performance. A number of reasons may account for these results. First, the visual search task we employed was specifically designed to test the participants’ ability to integrate global features^19,20,21,22^ as we solely induced positive emotion. The stimuli themselves as well as the number of stimuli is thus different to the global–local visual attention tasks previously used^16^. We can only speculate whether these differences led to varying degrees of complexity, which in turn not only tapped visual attention but also further cognitive domains. The relatively large number of erroneous and null responses may substantiate this assumption. We strongly encourage future research to use tasks, which both differentiate between perceptual and cognitive processes and further address different levels of complexity when investigating the effect of humor on cognition and perception. Second, with the humorous clip being rated funnier than the control clip, the present study was able to successfully induce humor in one group of participants. However, an exploratory analysis of individual funniness ratings within this humor group showed that the funnier these individuals rated the movie, the less accurate they performed in the visual search task. Interestingly, this finding is contrary to our predictions and leaves ample room for speculation. For instance, individuals that rated the movie clip as funnier, may have engaged more strongly with the film, thus showing a higher motivational intensity^32^, a concept closely related to valence and arousal^33^. As has been argued for humor and pain, it may be the level of arousal that drives the distraction effect^9^. Consequently, a comparable mechanism may account for the observed relationship between funniness ratings and accuracy rates within the humor group of the present study. Similarly, the higher the motivational intensity, the narrower the scope of attention^32^, which in turn would promote rather local than global processing strategies for those most involved. In fact, this is the pattern, we observed within the humor group. However, it needs to be pointed out, that at the group level no such differences were found, neither for accuracy rates nor on response times. Whether the result of our exploratory analysis accounts for a genuine or rather spurious effect remains an open question and demands further investigation. Likewise, the roles of motivational intensity and arousal, respectively, deserve additional attention. Finally, due to technical failure, all analyses regarding psychological stress were carried out with only 39 participants. Clearly, a larger sample may have yielded different results.

In conclusion, the present study clearly demonstrates the beneficial effects of a short humorous intervention on the psychological and physiological perception of subsequent stressful event. To our knowledge, it is the first study to have done so, extending findings from stress responses relating to the nervous as well as the immune system to those of the endocrine system. Yet, the exact nature of how humor affects psychological and physiological stress in detail are still subject to further research. While being beneficial for attenuating stress and cortisol levels, humor was found to have had no impact on cognitive performance, specifically on visual attention. On the surface, this may contradict assumptions of the broaden-and-build theory, yet results of an exploratory analysis point towards influential roles of motivational intensity and arousal, respectively. Taken together, a short humorous intervention can be a powerful instrument, improving our mental and physical health—not just on the way to the job interview but in many other situations of everyday life.

## Methods

### Participants

Of the 79 healthy participants tested (55 female; mean age = 24.1 years, *SD* = 5.09), 41 were randomly assigned to the humor condition (30 female; mean age = 25.1 years, *SD* = 5.44) and 38 to the control condition (25 female; mean age = 23.1 years, *SD* = 4.51). Participants received either 12 Euro or course credits in compensation. Prior to the experiment, written informed consent was obtained. The study was conducted in accordance with the Code of Ethics of the World Medical Association and approved by the ethics committee of the University of Lübeck. Testing took place at the Center of Brain, Behavior and Metabolism (CBBM) in Lübeck, Germany.

### Stimuli and apparatus

Participants in the humor group were shown movie clips depicting either harmless but funny mishaps of humans and animals while participants in the control group watched neutral interactions of humans and animals. The clips were displayed on a 17.30-in. screen (HP ProBook 470 G5, Hewlett-Packard Development Company, Palo Alto, CA, USA) with a resolution of 1600 × 900 Px. Saliva samples were collected via Salivette Cortisol (SARSTEDT AG & Co., Nümbrecht, Germany) and used to determine individual cortisol levels as a marker for physiological stress. Pain tolerance and subsequently stress levels were induced using a Digitimer DS5 Isolated Bipolar Constant Current Simulator (https://www.digitimer.com). The visual stimuli of the visual search task were presented with Matlab 2018b (https://www.Mathworks.com) using Psychtoolbox 3 (http://psychtoolbox.org/). Visual stimuli appeared in purple on a grey background and were displayed on a 27-inch CRT monitor (resolution: 1920 × 1080 Px, refresh rate: 120 Hz).

### Design and procedure

**Figure 4:**
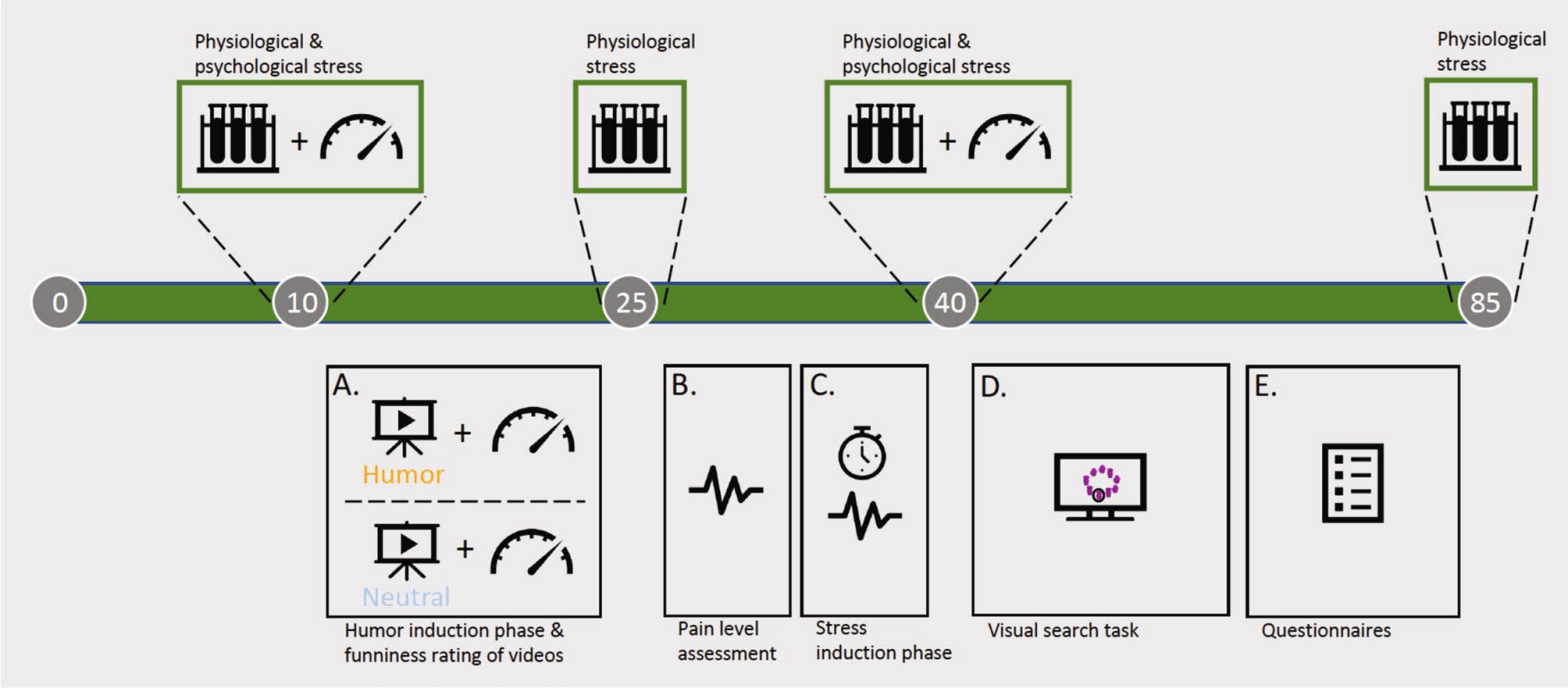
Study design. The upper half depicts the timepoints of salivary cortisol measurements and psychological stress ratings. The lower half shows the five phases of the experiment **(A–E)**. **(A)** Humor induction, **(B)** assessment of pain tolerance, **(C)** stress induction, **(D)** visual search task (cognitive performance), **(E)** self-assessment via questionnaires (Sense of Humor Scale and state and trait anxiety). Circled numbers denote the time in minutes from the beginning of the study. The figure was created using Microsoft PowerPoint for Mac (Version 16.43; www.microsoft.com).

The experiment involved five phases: humor induction, assessment of pain tolerance, stress induction, assessment of cognitive performance and self-assessments via personality questionnaires. Psychological stress was measured before humor induction and directly after stress induction by asking participants to rate their stress level on a scale from 1 to 10. Additionally, a total of four salivary cortisol samples were taken per participant: the first before the humor induction phase (T1), the second and the third after 15 and 30 min (T2 and T3), respectively, and the fourth after 75 min (T4; cf. Fig. 4). To control for the typical daily decline in cortisol levels, participants in both groups were on average tested at approximately the same time during the day.

In the first phase of the experiment, participants were randomly assigned to watch either the humorous movie clip (9 min 30 s) or the neutral one (9 min 33 s). Afterwards, participants were asked to rate the funniness of the clips on a 10-point scale (1 = *not at all funny*, 10 = *very funn*y) to check whether the films successfully induced a humorous mood. At the beginning of the second phase, we assessed the individual level of pain tolerance. For this purpose, an electrode was placed on the abductor pollicis brevis muscle of the participant’s left hand. The individual maximum level of pain tolerance was determined by applying a current of increasing intensity up to the point when the participant indicated it to be unpleasant. In the third phase of the experiment, the individual level of pain tolerance was used to support stress induction. For that purpose, each participant received randomly 30 mild shocks of individual intensity (up to 50 mA for max. 200 ms). In a five-minute stress test, participants were challenged to loudly count down from 500 in steps of seven. In case of making a mistake, they had to restart. To increase stress, participants had to perform this task in front of the experimenter, a clock counting down the time was in plain view and 20 randomly distributed shocks at the maximum level of pain tolerance were administered. The stress induction phase was followed by the visual search task. The visual search task consisted of four runs with 45 trials each. Trials were separated by jittered intervals of 500 to 1000 ms. For each trial, a circle of nine Greebles^19^ was presented for 5 s or until the participant’s response. Each circle consisted of two sets of four identical Greebles and one Greeble being different to all other Greebles. Participants had to press the spacebar as soon as they identified the odd Greeble out and click on it using a mouse. The position of the odd Greeble as well as the kind of Greebles shown varied randomly from trial to trial. Response times and accuracy rates were obtained to evaluate the effects of humor and stress induction. Finally, at the end of the experiment, participants filled out questionnaires regarding their sense of humor (Sense of Humor Scale)^24^ and their state and trait anxiety (STAI)^23^. The entire experimental session lasted for approx. 90 min.

### Data analyses

Funniness ratings and questionnaire data were analyzed using nonparametric tests for independent samples (Wilcoxon rank-sum test) as they were measured on an ordinal scale, were non-normally distributed and had unequal variances. For the same reason, correlations between funniness ratings and other variables were calculated using Kendall’s tau. To assess the level of psychological and physiological stress as a function of time and group, a mixed effect ANOVA was performed for which time served as a within and group as a between subject factor. We included the interaction of both variables, as well as an error term to account for between subject variance across the within subject variable. The actual change of psychological stress was determined by subtracting stress ratings before the stress test from those after the test and subjecting these values to an independent t-test with group as the grouping factor. We assessed the change over time (T1—T4) in physiological stress levels by calculating the area under the curve with respect to increase (AUC_I_) as proposed by Pruessner and colleagues^26^. Two further independent t-tests were used to examine whether changes in physiological stress levels and the level of pain tolerance differed between participants in the humor compared to the control group. The effect of humor and stress on cognitive performance in the visual search task was calculated by conducting an independent t-test for response times and a Wilcoxon rank-sum test for the (nonparametric) accuracy data. Before subjecting RT and accuracy data to analyses, we had to exclude one subject as there were no data recordings available. Additionally, missing responses (6.77% of the data) were classified as incorrect, resulting in a total of 15.3% of incorrect responses. Furthermore, we excluded RTs shorter than 100 ms assuming an anticipated rather than a true response (0.1% of the data), responses three SDs above or below the mean RT per participant (two data points) as well as participants with more than 40% of erroneous responses (4 subjects; cf. ^34,35^ for selection criteria/cut-offs). Finally, we performed two regression analyses to assess the (combined) effects of changes in psychological stress and changes in cortisol levels on cognitive performance (response times and accuracy). All data were analyzed in R-Studio^36^ with the exception of the Bayesian analyses, which were performed using JASP^37^. ANOVAs were calculated using the *afex* package, which corrects the degrees of freedom for repeated-measures factors with more than two levels^38^. Plots were created according to the tutorial paper by Allen and colleagues^39^.

## Data Availability

The data is available from the corresponding author upon request.
